# Construction of Uniform LiF Coating Layers for Stable High-Voltage LiCoO_2_ Cathodes in Lithium-Ion Batteries

**DOI:** 10.3390/molecules29061414

**Published:** 2024-03-21

**Authors:** Ziyang Xiao, Xiangbing Zhu, Shuguang Wang, Yanhong Shi, Huimin Zhang, Baobin Xu, Changfeng Zhao, Yan Zhao

**Affiliations:** School of Energy and Mechanical Engineering, Dezhou University, Dezhou 253023, China

**Keywords:** LiCoO_2_, LiF nanoshells, coating layers, high-voltage

## Abstract

Stabilizing LiCoO_2_ (LCO) at 4.5 V rather than the common 4.2 V is important for the high specific capacity. In this study, we developed a simple and efficient way to improve the stability of LiCoO_2_ at high voltages. After a simple sol–gel method, we introduced trifluoroacetic acid (TA) to the surface of LCO via an afterwards calcination. Meanwhile, the TA reacted with residual lithium on the surface of LCO, further leading to the formation of uniform LiF nanoshells. The LiF nanoshells could effectively restrict the interfacial side reaction, hinder the transition metal dissolution and thus achieve a stable cathode–electrolyte interface at high working-voltages. As a result, the LCO@LiF demonstrated a much superior cycling stability with a capacity retention ratio of 83.54% after 100 cycles compared with the bare ones (43.3% for capacity retention), as well as high rate performances. Notably, LiF coating layers endow LCO with excellent high-temperature performances and outstanding full-cell performances. This work provides a simple and effective way to prepare stable LCO materials working at a high voltage.

## 1. Introduction

With the development of clean energy, lithium-ion batteries, as a power storage device, have attracted a lot of attention due to their relatively high energy densities, low self-discharge properties and long service life. During the past few decades, the cathode materials in lithium-ion batteries (LIBs), such as LiNi_1/3_Mn_1/3_Co_1/3_O_2_ (NMC), LiNi_0.8_Co_0.15_Al_0.05_O_2_ (NCA) and LiFePO_4_ (LFP), have made significant progress and are widely applied in the electric vehicle sectors and energy storage devices [1,2,3,4]. Typically, LiCoO_2_ (denoted as LCO) has a high compacted density, resulting in a higher volumetric energy density compared to the above cathode materials [5,6,7]. Therefore, it has been extensively utilized in consumer electronics. However, LCO has a limited theoretical specific capacity of 274 mAh·g^−1^, which is much smaller than that of graphite anodes (372 mAh·g^−1^) [8,9,10]. Worse still, LCO usually works with a limited cut-off voltage, typically under 4.2 V (vs. Li/Li^+^), and thus demonstrates a low specific capacity of ~140 mAh·g^−1^ [11,12,13]. This situation indicates that it is crucial to enhance the energy density of lithium cobalt oxide. It would open up possibilities for lighter, thinner and longer-lasting consumer electronic products.

It is well known that raising the working voltage could effectively increase the energy density of LCO materials. However, there is the risk of structural degradation as well as more unstable cathode surfaces [14,15,16]. Under high pressure, lithium cobalt oxide is often accompanied by irreversible and detrimental phase transitions starting from the surface. The internal stress changes caused hinder the lithium-ion transport in the materials, showing poor cycling performances and rate capabilities [17,18,19,20]. Furthermore, the higher delithiated LiCoO_2_ (more than 0.5 Li removed at a cut-off voltage above 4.2 V) is highly oxidized, which could increase the likelihood of side reactions between the electrode materials and the electrolyte [21,22,23]. The resulting Co metal dissolution, electrolyte decomposition and surface phase degradation lead to poor cycling stability and safety problems [24,25,26]. Based on the above points, developing an effective strategy for stabilizing LCO, especially the surface stability of materials at high voltage windows, is crucial to achieve high cycling stability and enhance the specific capacity.

Coating and doping are regarded as two major modification methods to enhance the electrochemical performance of electrode materials. Doping can alter the microstructure and suppress the phase transition in electrode materials. Also, it carries the risk of reducing the specific capacity of electrode materials due to the introduction of non-electrochemically active cations [27,28]. Constructing coating layers has been demonstrated as an effective strategy to obtain excellent electrochemical properties of LCO at high working voltages [29,30]. Specifically, coating layers could prevent the contact between LCO and electrolytes, thereby suppressing surface side reactions and promoting a stable surface structure, achieving excellent stability [31,32]. The past few decades have witnessed enormous progress in coating layers, such as metal oxides, metal fluorides and polymers [28,33,34,35,36,37]. However, many surface problems needing to be resolved still exist. Typically, some oxide coatings cannot resist the erosion of hydrofluoric acid originating from the decomposition of electrolytes during the battery cycling process. In other words, oxides react with hydrofluoric acid, leading to the destruction of the coating [38]. Metal fluorides, able to resist HF, are a better choice for coating layers. Some metal fluorides, such as AlF_3_, MgF_2_ and other species, are also used as coating layers [39,40,41,42,43]. However, many metal fluorides cannot conduct lithium ions and electrons, thus adversely affecting the battery’s kinetic performance. Based on the excellent lithium-ion conductivity, LiF is considered the most promising coating material. Many efforts have been made to construct lithium fluoride coating layers [37,44,45,46,47]. Unfortunately, the usual utilizations of solid-phase precursors make it difficult to achieve uniform coating. Furthermore, many aqueous synthesis schemes have no consideration of the surface residue formation between LCO and water, such as LiOH and Li_2_CO_3_ [48,49], which could degrade the electrochemical performance. Therefore, it is necessary to develop a new method to construct a uniform lithium fluoride coating layer and eliminate the influence of residual lithium on the surface.

Herein, uniform LiF coating layers are constructed on the surface of LCO through a simple and efficient way. Specifically, trifluoroacetic acid was introduced to the LCO with residual lithium through the sol–gel method. High temperatures facilitated the reaction between the trifluoroacetic acid and residual lithium to form LiF coating layers, which not only remove the harmful residual lithium on the surface, but also ensure the uniformity of the lithium fluoride coating layer due to the uniform surface residual lithium. As a consequence, benefiting from high Li^+^ conductivity and the protection of LiF coating layers, the LCO@LiF exhibited greatly enhanced cycling stabilities and higher rate performances. More importantly, the LiF coating layers endowed LCO with a high cycling stability even charged to 4.5 V. Specifically, the capacity retention of LCO@LiF was 83.54% after 100 cycles, while the bare LCO only maintained 43.3% of the initial capacity. The LCO@LiF could deliver 140.2 mAh·g^−1^ at 5 C, which is much higher than bare LCO (108.5 mAh·g^−1^). Moreover, the LCO@LiF exhibited favorable high-temperature properties and achieved excellent cycling stabilities in full-cell scenarios. In addition, we identified that excessive LiF on the surface could cause poor electrochemical properties in LCO. Above all, our method provides a facile way to construct LiF coating layers on LCO surfaces, which exhibits a great potential in high-voltage LCO materials.

## 2. Results and Discussion

**Scheme 1:**
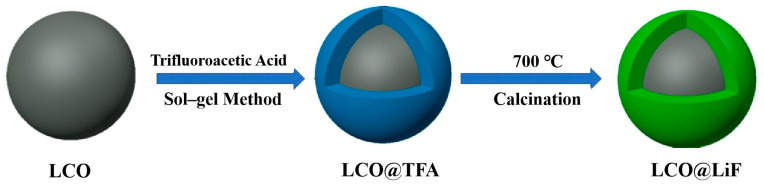
The schematic diagram for the preparation of LCO@LiF.

**Figure 1:**
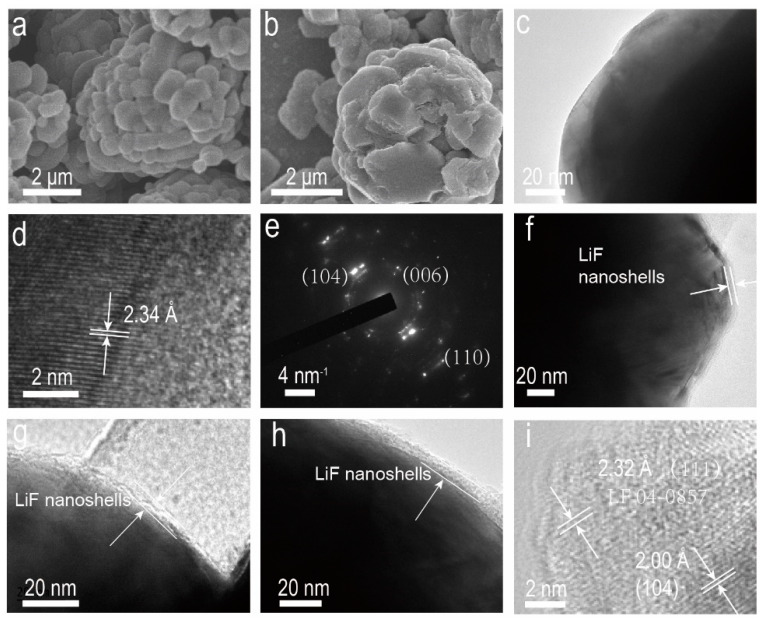
SEM images of (**a**) LCO and (**b**) LCO@LiF. (**c**) TEM and (**d**) HR−TEM images of LCO. (**e**) SAED image of LCO. (**f**–**h**) TEM and (**i**) HR−TEM images of LCO@LiF.

The construction of LiF coating layers includes three steps (Scheme 1). Firstly, the LCO was synthesized according to the reported literature [50]. During the synthesis process, the lithium-containing precursor was excessive to ensure the generation of residual lithium on the surface, which could be verified by the XPS data later. Secondly, TFA was introduced to the surface of LCO through a typical sol–gel method. Finally, the LCO with TFA species was calcinated in air to prepare LCO with 1 wt% LiF (denoted LCO@LiF). The F-containing TFA released F element and reacted with the residual lithium on the surface to generate LiF nanoshells in the calcination process [51]. A scanning electron microscope (SEM) was utilized to characterize the samples during the synthesized process. As shown in Figure 1a, the synthesized LCO exhibits irregular spheres with primary particles of around 2 μm. After coating LiF, the LCO samples still have almost the same morphology (Figure 1b), which indicates that the coating process did not cause an adverse effect to the samples’ morphology. Compared with the particle size and specific surface area of the above two samples (Table S1), the size of the coating layer is almost negligible, indicating that the change in the specific surface area of the material due to coating is extremely small. To further reveal the difference before and after LiF coating, a transmission electron microscope (TEM) was applied to observe the samples. As shown in Figure 1c, the bare LCO exhibits a smooth surface, indicating no surface modification. The high-resolution transmission electron microscope (TEM) reveals that the lattice spacing of the material is 2.34 Å (Figure 1d), matching the interplanar spacing of the (106) plane in lithium cobalt oxide. The selected area electron diffraction (SAED) image (Figure 1e) reveals the crystal planes, (111), (102), and (235), of lithium cobalt oxide, which suggests the existence of crystal grains with varied orientations within the material. This further confirms that the synthesized material is lithium cobalt oxide. The aforementioned findings will be verified through XRD patterns in the subsequent discussion.

After the coating process, the morphology of the material is shown in Figure 1f–h. It can be observed that the surface of lithium cobalt oxide is covered with a lithium fluoride coating layer. High-resolution transmission electron microscopy was employed to analyze the physicochemical properties of the material. As shown in Figure 1, the lattice spacing in the electrode material is 2 Å, corresponding to the (104) crystal plane of the LCO materials. Within the coating layer, it is observable that the crystal plane has a lattice spacing of 2.32 Å, aligning with the (111) crystal plane of lithium fluoride. This serves as evidence for the existence of lithium fluoride. Evidence for the presence of lithium fluoride via XRD and XPS will be provided in the later discussion.

**Figure 2:**
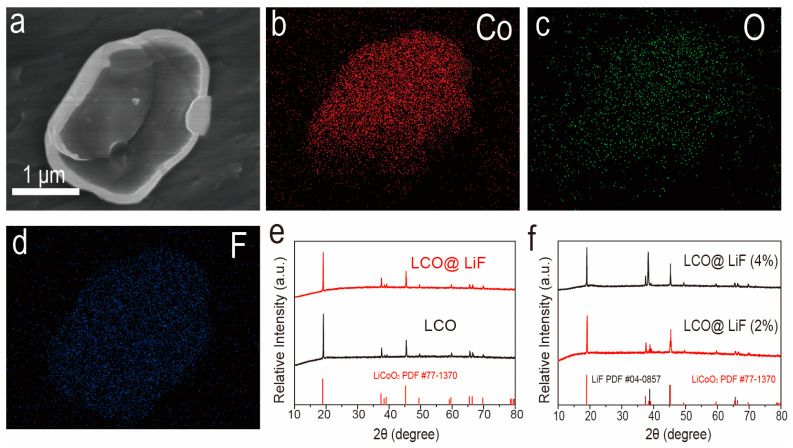
(**a**) SEM image of a randomly picked particle. Element mapping of the (**b**) Co element, (**c**) O element and (**d**) F element. (**e**) XRD patterns of LCO and LCO@LiF. (**f**) XRD patterns of LCO@LiF (2 wt%) and LCO@LiF (4 wt%).

An Energy Dispersive Spectrometer (EDS) was utilized to explore the distribution of LiF coating layers. As shown in Figure 2a−d, the O and F elements distribute evenly on the surface of LCO, which agrees with the location of Co elements. This demonstrates the uniformity of the LiF nanoshells. The Energy Dispersive X−ray Spectroscopy (EDX) result indicates that the contents of F reach about 0.76% (Figure S1). Also, X-ray diffraction (XRD) was conducted to further explore the LCO change before and after the coating treatment. As shown in Figure 2e, the XRD patterns of LCO and LCO@LiF exhibit almost the same peaks, and they both match well with LiCoO_2_ (JCPDS No. 77-1370). The result further proves that our method did not cause adverse effects on LCO. The characteristic XRD peaks of LiF were not observed in the LCO@LiF patterns, which is probably due to the tiny amount of LiF. To further demonstrate the existence of LiF species, the LCO materials with more LiF species (2 wt% and 4 wt%) were characterized (Figure 2f). It was found that the XRD results of LCO@LiF (2%) and LCO@LiF (4%) exhibit the characteristic peaks of LCO and LiF, indicating the presence of LiF originating from the reaction between LCO and TFA. Also, as the amount of trifluoroacetic acid (TFA) increases, the intensity of the characteristic peaks of lithium fluoride rises. This is attributed to the reaction between more TFA and lithium cobalt oxide, which leads to the generation of more LiF.

**Figure 3:**
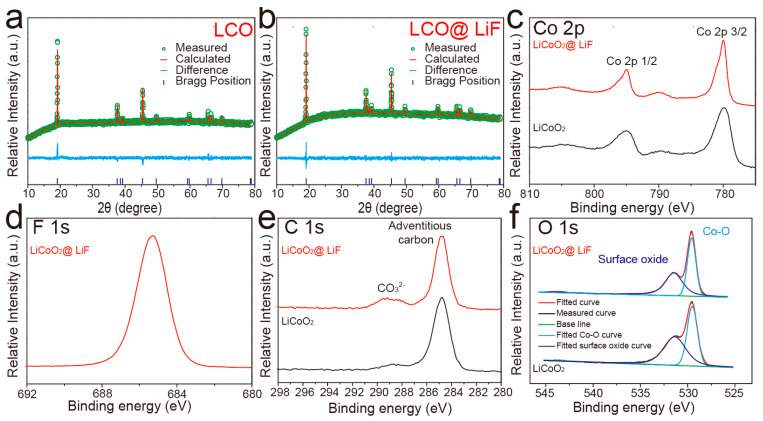
Rietveld refined XRD patterns of (**a**) LCO and (**b**) LCO@LiF. High−resolution XPS spectra of elements in LCO@LiF and LCO: (**c**) Co 2p, (**d**) F 1s, (**e**) C 1s and (**f**) O 1s.

To further reveal the influence of the LiF coating on the cell parameters of LiCoO_2_, the Rietveld refinement was applied to the LCO and LCO@LiF (Figure 3a,b). The refined XRD results are presented in Table 1. It is evident that the refined wR values are 2.585% and 2.147%, respectively, suggesting that the refined outcomes are reasonable and within acceptable limits. The I(003)/I(104) value and (I(006) + I(102))/I(101) value of LCO are almost the same as those of LCO@LiF, indicating the nearly identical Li/Co anti-site and hexagonal order. The c/a ratio of the electrode material remains around 4.993 before and after coating. Moreover, the unit cell parameters of lithium cobalt oxide and lithium cobalt oxide coated with lithium fluoride are nearly identical, indicating that the LiF coating treatment did not impose any detrimental effects on the lattice structure. For example, the c lattice parameter in the LCO cell is 14.062 Å, while the c lattice parameter of LCO@LiF is 14.056 Å. And the values in the unit cells of lithium cobalt oxide and lithium cobalt oxide coated with lithium fluoride are both around 2.81 Å, indicating no significant changes. All the results suggest the LiF coating process did not cause detrimental effects on the crystal structure of the LCO material.

X-ray photoelectron spectroscopy (XPS) of LiCoO_2_@LiF and LCO was also conducted to investigate the valence of surface elements. As demonstrated in Figure S2, the survey scan XPS spectra of LCO have signals of Li, Co, O and C, which come from the LiCoO_2_ materials and absorbed carbon. The LCO@LiF exhibits the existence of Li, Co, O and F, proving the existence of LiF nanoshells. High-resolution XPS spectra of Co 2p, F 1s, C 1s and O 2p were also collected. As shown in Figure 3c, the LCO has almost the same Co 2p spectra as the LCO@LiF, indicating that the coating process has almost no effect on the chemical bonding of Co. Specifically, the Co spectra of LCO and LCO@LiF both have two main peaks relative to Co 2p_3/2_ and Co 2p_1/2_ [37,52,53]. Furthermore, the two main peaks can be deconvoluted into four subpeaks (Figure S3). Specifically, for LCO@LiF, the subpeaks around 779.8 eV and 794.6 eV are relative to Co^3+^, while the subpeaks around 781.32 eV and 796.15 eV correspond to Co^2+^. For LCO, the subpeaks of Co^3+^ are located at 779.82 eV and 794.89 eV, while the peaks of Co^2+^ appear at 780.76 and 796.24 eV [52,53]. The small binding energy shift between the two samples should be ascribed to the changed chemical environment of Co in LCO and LCO@LiF. As for the F element (Figure 3d), there exists a main peak at 685 eV in the F 1s spectra of LCO@LiF, indicating the existence of LiF [53]. In addition, the C 1s spectra exhibits two main peaks located at 289.6 eV and 284.5 eV (Figure 3e), corresponding to carbonate species of Li_2_CO_3_ and adventitious carbon [51,52,53,54]. The LCO@LiF exhibits a low peak intensity of carbonate species of Li_2_CO_3_, indicating that our method decreased the residual lithium on the surface of LCO. The result implies that the lithium fluoride coating layer is derived from the reaction between TFA and surface residual lithium on the surface of LCO, confirming our speculation. The O 1s spectra shows two main peaks at 529.39 eV and 531.24 eV (Figure 3f), corresponding to Co-O and absorbed OH^−^ [55,56]. After the coating treatment, the peak at 531.24 eV decreases slightly, which may be ascribed to the reduced residual lithium, agreeing with the C 1s spectra. Above all, the XPS results further demonstrate that our method has a positive effect on LCO when the LiF species exists on the surface.

**Figure 4:**
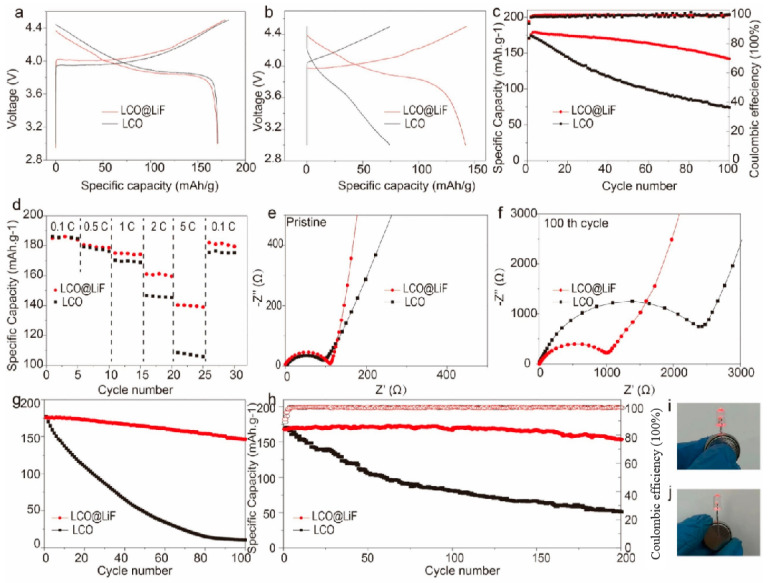
Electrochemical performance of both pristine LCO and LCO@LiF in half−cell scenario: (**a**) 1st charge/discharge curves, (**b**) 100th charge/discharge curves, (**c**) cycling stability test 0.5 C, (**d**) rate performance testing from 0.1 C to 5 C, (**e**) electrochemical impedance of the pristine samples and (**f**) electrochemical impedance of the samples after 100 cycles. (**g**) Cycling stability at 1 C in 60 °C. Electrochemical performance of both pristine LCO and LCO@LiF in full−cell scenario: (**h**) cycling stability at 1 C, (**i**) image of lighting an LED with a full cell. (**j**) Image of the LED light after 3 days of operation with a full cell.

It is well known that LiF could be regarded as a good protective coating layer with high lithium-ion conductivity, which inspired us to explore its application in lithium-ion batteries. Figure 4a shows the first charge/discharge curves of the LCO and LCO@LiF in half-cell scenarios between 3 and 4.5 V. The first discharge specific capacity of the LCO@LiF and LCO was 170.1 mAh·g^−1^ and 170.9 mAh·g^−1^, respectively, indicating that the LiF nanoshells did not have a negative impact on the capacity. The curve shapes have nearly no difference between the two curves in the first cycle, indicating that the LiF nanoshells have no adverse impact on the electrochemical properties. Notably, the coulombic efficiency of the LCO@LiF (95.28%) is higher than that of the LCO (93.81%), implying that the LiF nanoshells could hinder the side reaction. When the cycling number increases, the LCO@LiF exhibits superior discharge specific capacity than the bare LCO. For example, LCO exhibits the specific discharge capacity of 174.5 mAh·g^−1^ at the 20th cycle, which is higher than the bare LCO (144.8 mAh·g^−1^) (Figure S4). At the 50th cycle, the LCO@LiF (166.9 mAh·g^−1^) still exhibits advantages over bare LCO (107.2 mAh·g^−1^) in the discharge specific capacity (Figure S5). After 100 cycles (Figure 4b), the bare LCO exhibits severe voltage decay compared with the LCO@LiF, confirming the superiority of LiF nanoshells. Figure 4c compares the cycling stability of LCO and LCO@LiF. Specifically, the LCO@LiF exhibits 142.1 mAh·g^−1^, corresponding to 83.54% of the initial discharge capacity, while LCO only retains 43.3% of the initial discharge capacity. In the dQ/dV curve (Figures S6 and S7), during the cycling process, the peak shift of lithium cobalt oxide (LCO) is noticeably larger than that of lithium cobalt oxide coated with lithium fluoride (LCO@LiF), suggesting significant polarization and the occurrence of irreversible phase transitions and energy losses in the lithium cobalt oxide material during the cycling process [57]. This result strongly demonstrates the advantages of LiF coating layers. Compared with other reported LCO-based samples, our LCO@LiF exhibits certain advantages in electrochemical performance (Table S2).

To further reveal the mechanism behind the superior performance of LCO@LiF, the transition metal dissolution of electrode materials after 100 cycles was tested through inductively coupled plasma atomic emission spectroscopy (ICP-MS). It was found that the LiF nanoshells greatly reduce the amount of Co^3+^ dissolved in the electrolyte (Table S4), which explains the advantages of LiF nanoshells. Moreover, the TEM image (Figure S8) shows that the cycled LCO@LiF still maintains a good surface coating layer after cycling. In addition, the electrochemical performance of LCO with more LiF nanoshells was also explored. As shown in Figure S9, the discharge specific capacity of LCO with 2 wt% LiF and 4 wt% LiF was reduced to 161.9 mAhg^−1^ and 107.6 mAhg^−1^, maybe due to the existence of more inactive LiF species and the destruction of the LCO structure by excessive TFA [58,59].

The rate performance of the LCO@LiF and LCO was also investigated. As shown in Figure 4d, LCO@LiF exhibited 140.2 mAh·g^−1^ at 5 C, while the LCO only delivered 108.5 mAh·g^−1^. This result confirms the elevated transportation dynamics of Li^+^. Moreover, the charge-transfer impedance of the cathode samples was also tested before and after cycling. As shown in Figure 4e,f, the LCO and LCO@LiF exhibit similar high-frequency semicircles, demonstrating similar charge-transfer impedance before cycles. However, after 100 cycles, the LCO@LiF exhibits much higher resistance than the bare LCO, which hints that the high Li^+^ conductivity was brought by LiF and agrees with the result of Figure 4d [60]. Figure S10 presents the corresponding equivalent circuit, depicting R_CEI_ as the resistance encountered by Li^+^ during its passage through the cathode–electrolyte interphase, while R_ct_ denotes the resistance associated with charge transfer. The surface treatment resulted in a significant reduction in the values of both R_CEI_ and R_ct_, as compared to those exhibited by LCO (Table S3). All the above results clearly confirm that the LiF nanoshells enhanced the Li^+^ conductivity and endowed LCO with enhanced structural integrity for superior cycling stability at a high voltage of 4.5 V. Moreover, the high-temperature performance tests demonstrate that the LiF coating can assist LCO in maintaining excellent cyclic stability under harsh conditions (Figure 4g). Specifically, at a 1 C discharge rate, LCO@LiF can retain 83.29% of its initial specific capacity after 100 cycles, while uncoated lithium cobalt oxide only retains 19.3%. 

Moreover, the electrochemical performances of LCO@LiF obtained at 600 °C and 800 °C (referred to as LCO@LiF-600 and LCO@LiF-800, respectively) were also explored. Relative to the first cycle, the LCO@LiF-600 sample exhibits a capacity retention rate of 68.46% after 100 cycles (Figure S11), which may be attributed to the unfavorable conditions for the generation of the coating layer at this temperature [60,61]. As a comparison, the LCO@LiF-800 exhibits a capacity retention rate of 78.21% after 100 cycles (Figure S11), which is greater than that of LCO@LiF-600 (68.46%) but less than that of samples obtained at 700 °C (83.54%), possibly due to the reduced coverage efficiency of the LiF coating layer formation at higher temperatures [62]. Furthermore, we tested the rate performance of the materials at 5 C (Figure S12). The results reveal that the discharge specific capacity of LCO@LiF-600 (111.6 mAh·g^−1^) at 5 C is lower than that of LCO@LiF-800 (125.2 mAh·g^−1^), both of which are lower than that of LCO@LiF (140.2 mAh·g^−1^). This suggests that 700 °C is the optimal synthesis temperature for the LCO@LiF.

In addition, a full battery was tested with LCO as the positive electrode and prelithiated graphite as the negative electrode under a positive-to-negative ratio of 1:1.1 (Figure 4h, Figures S13 and S14). The test results indicate that the capacity retention of lithium cobalt oxide coated with lithium fluoride is 91.36% after 200 cycles at 1 C, which is significantly higher than the uncoated electrode material (30.28%). Furthermore, the full LCO/graphite battery can illuminate LED lights in children’s toys for a long time (Figure 4i,j), demonstrating the practicality of the LCO@LiF cathodes. 

## 3. Experimental Section

### 3.1. Synthesis of LCO

LCO was synthesized through a sol–gel method from the reported literature [50]. Namely, lithium acetate and cobalt acetate were added to 30 mL water with a molar ratio of Li: Co at 1.05:1. After stirring for 0.5 h, acrylic acid (AA) was added to the mixture with the ratio of the total metallic ions charges/AA at 1:1. Afterwards, the system was heated at 75 °C until a purple gel was achieved. Finally, the gel was heated at 800 °C to obtain LCO.

### 3.2. Preparation of LCO @LiF

Two steps were needed for the preparation of LCO@LiF. Firstly, 7.31 μL trifluoroacetic acid (TFA) was mixed with 0.5 g LCO under vigorous stirring conditions. Meanwhile, the system was heated at 60 °C until the solvent was totally evaporated. Secondly, the TFA-modified LCO was heated at 700 °C for 2 h with a heating rate of 5 °C/min to obtain the final product, LCO@LiF, in which the weight percentage of lithium fluoride in the material was 1%.

LCO with more LiF species was produced for comparison. Namely, 14.6 μL TFA was utilized for the preparation of LCO@LiF (2 wt%) under the same other conditions as the process for producing LCO@LiF, and 29.2 μL TFA was used for the LCO@LiF (4 wt%).

### 3.3. Characterization 

Details are shown in the Supporting Information.

## 4. Conclusions

Above all, the cycling stability and rate performance of LCO were improved through a surface engineering method. Specifically, a simple sol–gel approach followed by calcination successfully converted the residual lithium to uniform LiF nanoshells on the surface of LCO. Comparing the electrochemical measurement and material characterization results, the improvement in LCO@LiF performance should be attributed to the following aspects. First, the LiF coating layer can resist the erosion of substances such as hydrofluoric acid in the electrolyte, stabilize the surface of the electrode material, and thereby improve the cycling performance of the electrode material. Secondly, the coating layer can prevent surface side reactions, suppress the leaching of transition metal ions, and maintain the stability of the electrode material structure. Finally, the lithium fluoride coating layer has good ion conductivity, which can improve the kinetic performance at the interface and thereby enhance the rate performance of the electrode material. Our method underscores the significance of surface engineering and paves the way for the development of highly stable cathode materials for high-voltage applications.

## Figures and Tables

**Table 1:** Lattice parameters of LCO@LiF and LCO obtained from Rietveld refinements.

Atom	Site	x	y	z	Occupancy
LCO@LiF (wR = 2.147% GOF = 1.11) Space group: R-3m Lattice parameters: a = b = 2.81492 Å, c = 14.05559 Å, V = 96.452 Å^3^					
Li	3a	0.00000	0.00000	0.50000	1
Co	3b	0.00000	0.00000	0.00000	1
O	6c	0.00000	0.00000	0.26997	1
LCO (wR = 2.585% GOF = 1.22) Space group: R-3m Lattice parameters: a = b = 2.81608 Å, c = 14.06196 Å, V = 96.575 Å^3^					
Li	3a	0.00000	0.00000	0.50000	0.991 (11)
Co	3b	0.00000	0.00000	0.00000	0.996 (9)
O	6c	0.00000	0.00000	0.26000	1

## Data Availability

Data are contained within the article and Supplementary Materials.

## Supplementary Materials

The following supporting information can be downloaded at: https://www.mdpi.com/article/10.3390/molecules29061414/s1, Figure S1. The EDX pattern of LCO@LiF. Figure S2. XPS survey scan of LCO and LCO@LiF. Figure S3. High-resolution XPS spectrum of Co in (a) LCO and (b) LCO@LiF. Figure S4. The charge/discharge curves of LCO@LiF at the 20th cycle. Figure S5. The charge/discharge curves of LCO@LiF at the 50th cycle. Figure S6. The dQ/dV curves of LCO@LiF in the voltage range of 3.0–4.5 V at 0.5 C and room temperature in half-cell scenarios. Figure S7. The dQ/dV curves of LCO in the voltage range of 3.0–4.5 V at 0.5 C and room temperature in half-cell scenarios. Figure S8. TEM image of the cycled LCO@LiF. Figure S9. The cycling performance of LCO with 2 wt% and 4 wt% LiF. Figure S10. The equivalent circuit models of the EIS fitting. Figure S11. Cycling stability of LCO@LiF-800 and LCO@LiF-600 at 0.5 C. Figure S12. Charge/discharge curves of LCO@LiF-800 and LCO@LiF-600 at 5 C rate. Figure S13. The charge/discharge curves of LCO@LiF at the 1st cycle in a full cell. Figure S14. The charge/discharge curves of LCO@LiF at the 200th cycle in a full cell. Table S1. The test results of basic physicochemical data of the LCO and LCO@LiF samples. Table S2. The comparison of LCO@LiF with some reported LCO materials. Table S3. Results of EIS fitting. Table S4. Co dissolution amount after cycling measured by ICP-AES (based on the active material mass). References [63,34,63,64,65,66,67,68,69,70] are cite in the Supplementary Materials.
