# Supplementary material for: Construction of Uniform LiF Coating Layers for Stable High-Voltage LiCoO2 Cathodes in Lithium-Ion Batteries
Source: Molecules. 2024 Mar 21;29(6):1414. doi: 10.3390/molecules29061414 (PMC10974255; doi:10.3390/molecules29061414)
Supplement: Supplementary file 1 [file molecules-29-01414-s001.zip › molecules-2922528-supplementary.pdf]

Construction of uniform LiF coating layers for stable high-voltage $LiCoO_2$ cathodes in Lithium-Ion Batteries

Ziyang Xiao, Xiangbing Zhu, Shuguang Wang, Yanhong Shi, Huimin Zhang, Baobin Xu, Changfeng Zhao * and Yan Zhao *

School of Energy and Mechanical Engineering, Dezhou University, DeZhou, Shandong, 253023, China

*Correspondence: Changfeng Zhao, 867713584@qq.com; Yan Zhao, dzuzhy@126.com

Experimental Section

2.3 Characterization

2.3.1 Assembly of lithium-ion batteries

To prepare the cathode electrode, PVDF (polyvinylidene difluoride), carbon black, and cathode material are mixed in a ratio of 8:1:1 with NMP (N-methylpyrrolidone). The mixture is then homogenized and coated onto aluminum foil. The coated foil is dried in an oven at 80 °C. After drying, the coated foil is sliced to obtain the cathode material. For the anode electrode, lithium foil and prelithiated graphite are used as negative electrode materials in half-cells and full-cells, respectively. And prelithiated graphite is prepared by direct contact of lithium foil with graphite. The electrolyte consists of a mixed solvent (ethylene carbonate (EC)/ethyl methyl carbonate (EMC)/fluoroethylene carbonate (FEC) in a volumetric ratio of 1:1:1) containing $LiPF_6$ (1 M). The cathode, anode, separator, and electrolyte are assembled in a glove box with the water and oxygen content below 1 ppm. The prepared battery is then placed on a LAND CT2001A battery measurement system for electrochemical performance testing. The cycling test of half-cell is conducted at a charge/discharge rate of 0.5 C (theoretical specific capacity of 190 mAh·g-1) within a charge/discharge voltage range of 3-4.5 V. And the operational voltage range for the full cell is 3-4.45 V. The EIS data was obtained with the frequency ranging from 100 KHz to 0.01 Hz.

2.3.2 Instruments

A transmission electron microscope (TEM, JEOL-2100F, Japan) with the working voltage of 200 kV were utilized to conduct TEM characterization, while a field-emission scanning electron microscope (SEM, JEOL 6701F, Japan) was used for the collection of SEM data. A Rigaku MiniFlex 600 X-ray diffractometer with Cu Kα radiation (λ= 0.154 nm) was utilized to collect the XRD data. Thermo Scientific K-Alpha equipment (Al Kα radiation) was utilized to obtain the XPS spectra of samples. A Shimadzutype instrument (ICPE-9000) was utilized to determine the transition metal dissolution in the electrolyte. For transmission electron microscopy (TEM) testing, the prepared materials were dispersed in ethanol by ultrasonication, and then the ethanol containing the dispersed materials was dropped onto a carbon support film for TEM testing. For X-ray photoelectron spectroscopy (XPS) analysis, the prepared materials were dried at 80 ℃ in a vacuum drying oven for 10 hours, and then the materials were subjected to XPS testing.

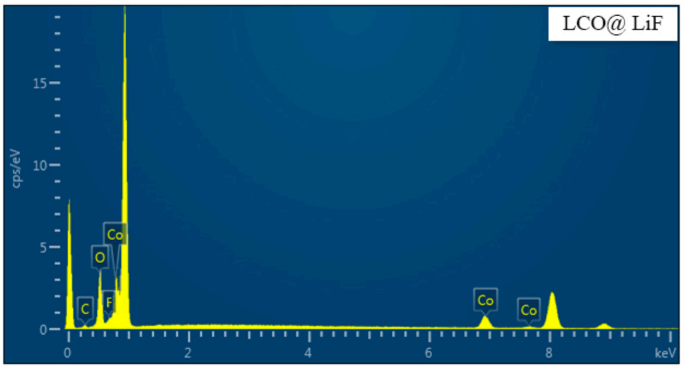

Figure S1. The EDX pattern of LCO@LiF.

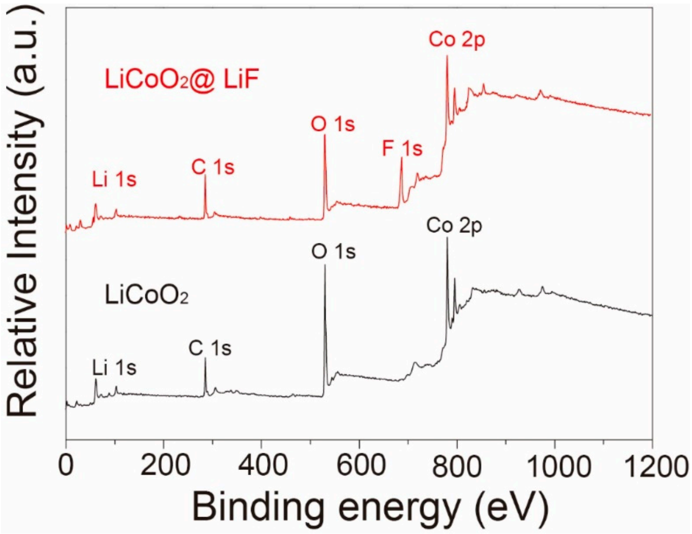

Figure S2. XPS survey scan of LCO and LCO@LiF.

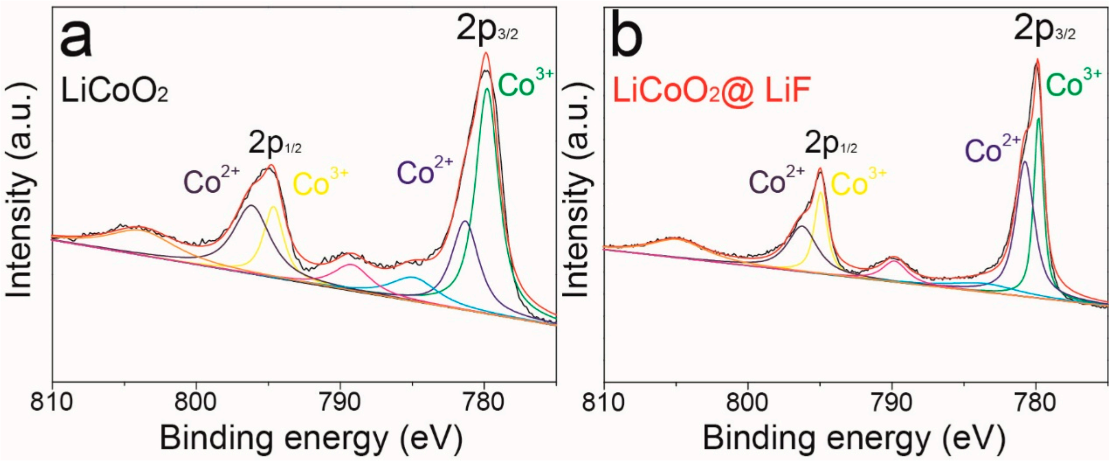

Figure S3. High-resolution XPS spectrum of Co in (a) LCO and (b) LCO@ LiF.

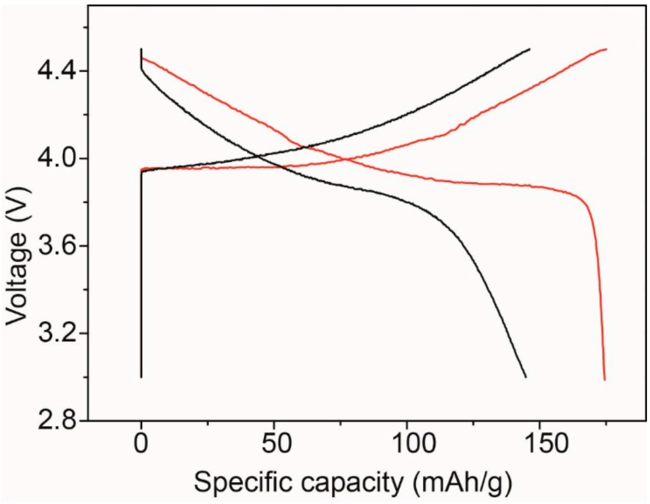

Figure S4. The charge/discharge curves of LCO@ LiF at 20 th cycle.

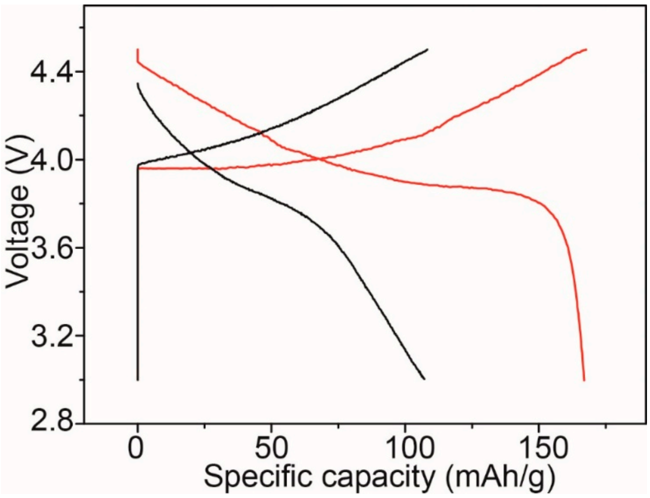

Figure S5. The charge/discharge curves of LCO@ LiF at 50 th cycle.

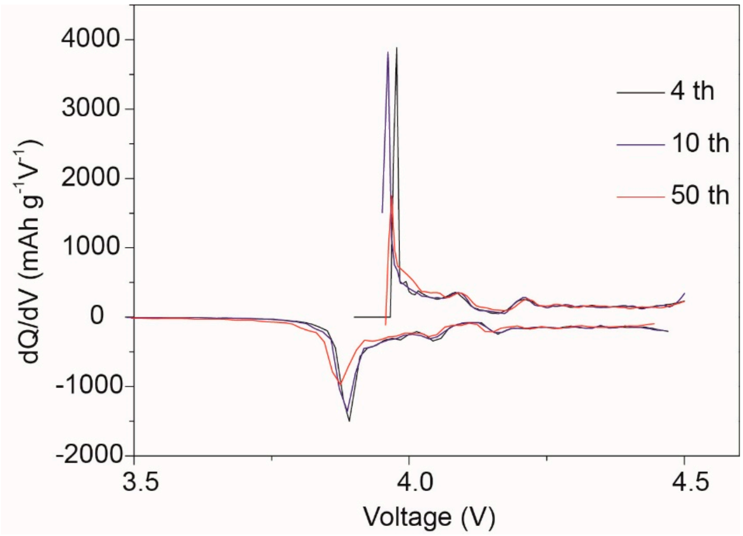

Figure S6. The dQ/dV curves of LCO@LiF in the voltage range of 3.0–4.5 V at 0.5 C and room temperature in half cell.

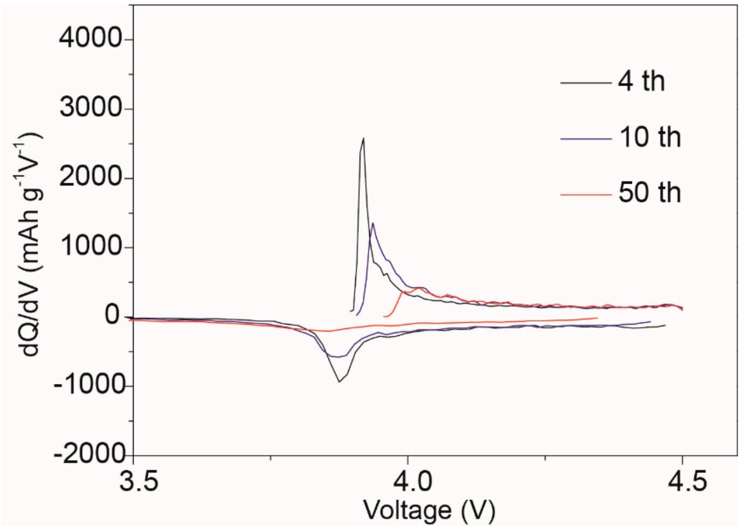

Figure S7. The dQ/dV curves of LCO in the voltage range of 3.0–4.5 V at 0.5 C and room temperature in half cell.

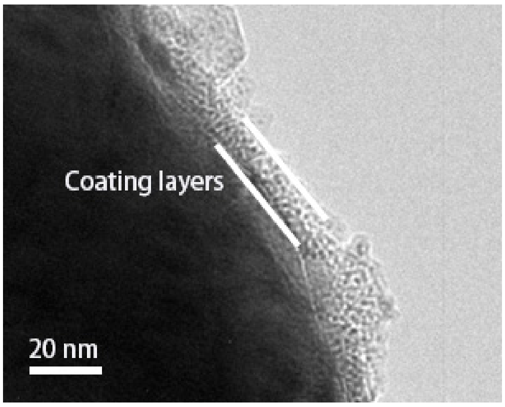

Figure S8. TEM image of the cycled LCO@LiF.

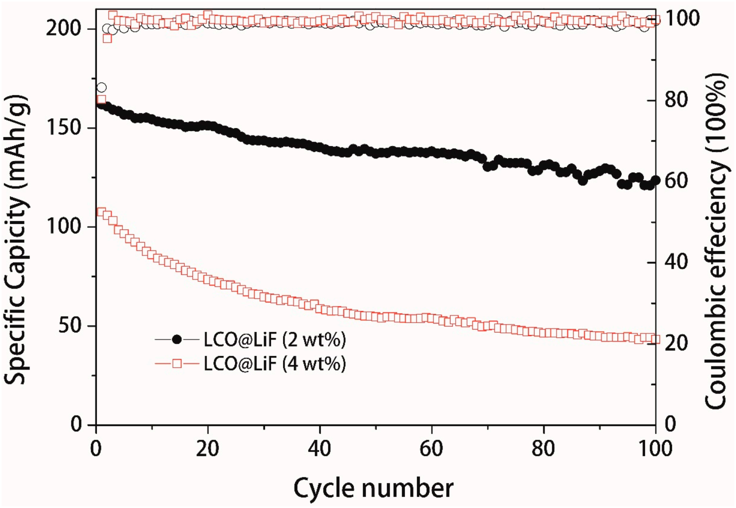

Figure S9. The cycling performance of LCO with 2 wt % and 4 wt% LiF.

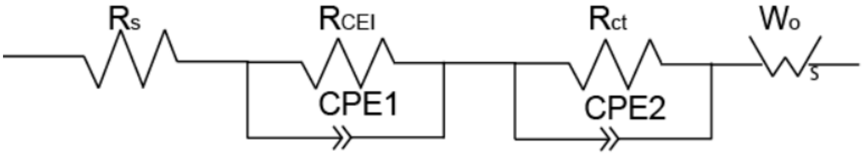

Figure S10. The equivalent circuit models of the EIS fitting.

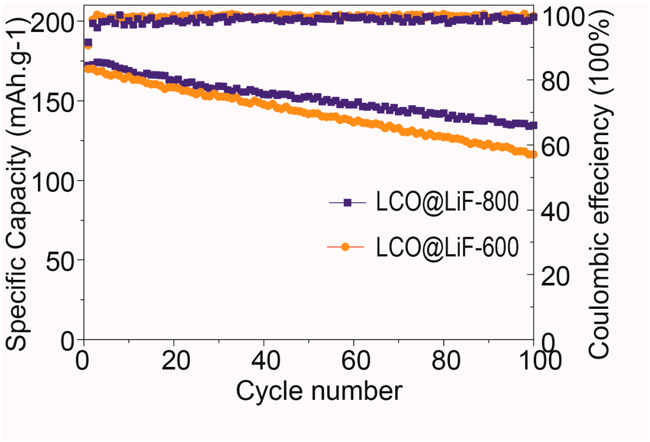

Figure S11. Cycling stability of LCO@LiF-800 and LCO@LiF-600 at 0.5 C

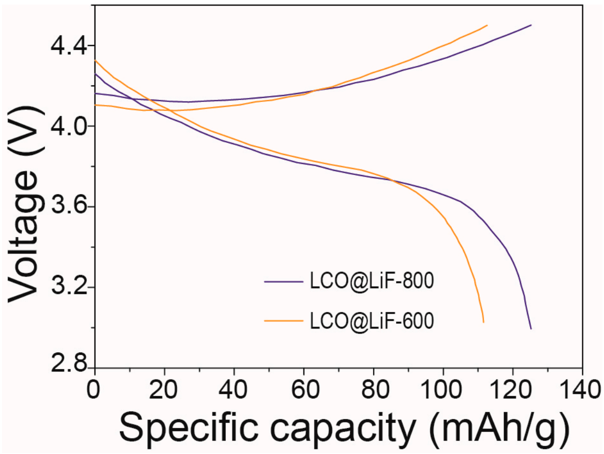

Figure S12. Charge/discharge curves of LCO@LiF-800 and LCO@LiF-600 at 5 C rate.

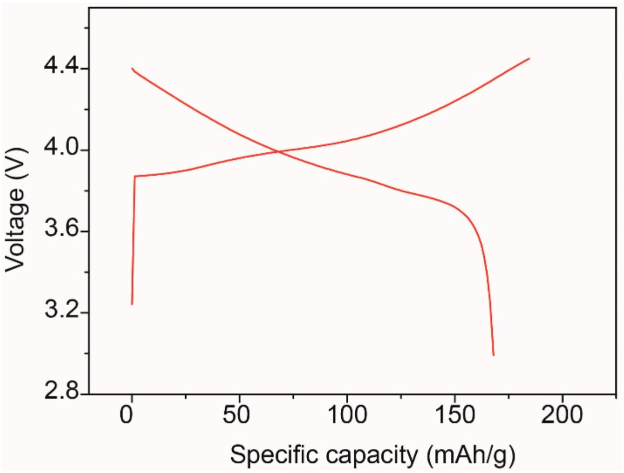

Figure S13. The charge/discharge curves of LCO@ LiF at 1 st cycle in the full cell

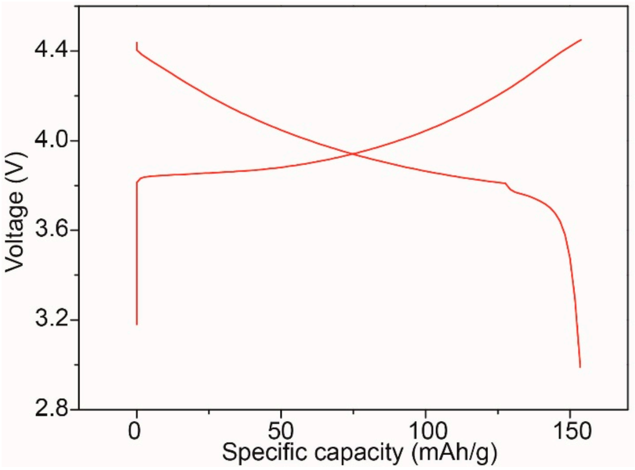

Figure S14. The charge/discharge curves of LCO@ LiF at 200 th cycle in the full cell

Table. S1 The test results of basic physicochemical data of the LCO and LCO@LiF samples

Sample	D10/μm	D50/μm	D90/μm	BET/$m^2 \cdot g^{-1}$
LCO	0.9	2.1	4.5	2.10
LCO@LiF	0.9	2.2	4.7	2.15

Table. S2 The comparison of LCO@LiF with some reported LCO materials

Modification methods	Cut-off voltage	Electrochemical performance	Ref.
LiF coating	4.5 V	83.54% retention after 100 cycles at 0.5 C	**Our work**
Mn_3O_4 coating	4.5 V	81.5% retention after 100 cycles at 1C	1
Li_3PO_4 coating	4.5V	79.3% retention after 100 cycles at 1C	2
$Li_4Ti_5O_{12}$ coting	4.5 V	90% retention after 60 cycles at 0.2 C	3
MgO coating	4.35 V	85% retention after 60 cycles at 0.2 C	4
$AlPO_4$ coating	4.5 V	75% retention after 160 cycles at 1 C	5
MgF_2 coating	4.5 V	80% retention after 50 cycles at 0.2 C	6
Cu doping	4.4 V	78.7% retention after 500 cycles at 2 C	7
Mg doping	4.5V	79% retention after 55 cycles at 1 C	8

W doping	4.6 V	72.3% retention after 100 cycles at 1 C	9
Bare LCO	4.5 V	55.2 % retention after 100 cycles at 1C	1

Table.S3 Results of EIS Fitting

Impedance	R_{CEI} (Ω)	R_{CT} (Ω)
LCO	22.5	81.4
LCO@ LiF	18.7	98.3
LCO (100 th)	268.9	2549.4
LCO@ LiF (100 th)	112.5	992.6

Table. S4 Co dissolution amount after cycling measured by ICP-AES (based on the active materials mass)

Dissolution amount	LCO	LCO@LiF
Co (wt%)	0.09	0.04

Reference

1. Wang, J.; Zhang, S. D.; Guo, S. J.; Lu, S. Q.; Xu, Y. S.; Li, J. Y.; Cao, A. M.; Wan, L. J. Stable 4.5 V $LiCoO_2$ Cathode Material Enabled by Surface Manganese Oxides Nanoshell. *Nano Res.* **2023**, *16*, 2480–2485, doi:10.1007/s12274-022-5010-2.

2. Zhou, A.; Xu, J.; Dai, X.; Yang, B.; Lu, Y.; Wang, L.; Fan, C.; Li, J. Improved High-Voltage and High-Temperature Electrochemical Performances of $LiCoO_2$ Cathode by Electrode Sputter-Coating with Li_3PO_4. *J. Power Sources* **2016**, *322*, 10–16, doi:10.1016/j.jpowsour.2016.04.092.

3. Zhou, A.; Dai, X.; Lu, Y.; Wang, Q.; Fu, M.; Li, J. Enhanced Interfacial Kinetics and High-Voltage/High-Rate Performance of $LiCoO_2$ Cathode by Controlled Sputter-Coating with a Nanoscale $Li_4Ti_5O_{12}$ Ionic Conductor. *ACS Appl. Mater. Interfaces* **2016**, *8*, 34123–34131, doi:10.1021/acsami.6b11630.

4. Shim, J. H.; Lee, S.; Park, S. S. Effects of MgO Coating on the Structural and Electrochemical Characteristics of $LiCoO_2$ as Cathode Materials for Lithium Ion Battery. *Chem. Mater.* **2014**, *26*, 2537–2543, doi:10.1021/cm403846a.

5. Cho, J.; Kim, T. G.; Kim, C.; Lee, J. G.; Kim, Y. W.; Park, B. Comparison of Al_2O_3 and $AlPO_4$-Coated $LiCoO_2$ Cathode Materials for a Li-Ion Cell. *J. Power Sources* **2005**, *146*, 58–64, doi:10.1016/j.jpowsour.2005.03.118.

6. Bai, Y.; Jiang, K.; Sun, S.; Wu, Q.; Lu, X.; Wan, N. Performance Improvement of $LiCoO_2$ by MgF_2 Surface Modification and Mechanism Exploration. *Electrochimica Acta* **2014**, *134*, 347–354, doi:10.1016/j.electacta.2014.04.155.

7. Kim, J.; Kang, H.; Go, N.; Jeong, S.; Yim, T.; Jo, Y. N.; Lee, K. T.; Mun, J. Egg-Shell Structured $LiCoO_2$ by Cu^{2+} Substitution to Li^+ Sites *via* Facile Stirring in an Aqueous Copper(II) Nitrate Solution. *J. Mater. Chem. A* **2017**, *5*, 24892–24900, doi:10.1039/C7TA07232E.

8. Yin, R. Z.; Kim, Y. S.; Shin, S. J.; Jung, I.; Kim, J. S.; Jeong, S. K. In Situ XRD Investigation and Thermal Properties of Mg Doped $LiCoO_2$ for Lithium Ion Batteries. *J. Electrochem. Soc.* **2012**, *159*, A253–A258, doi:10.1149/2.006203jes.

9. Zhang, J. N.; Li, Q. H.; Li, Q.; Yu, X. Q.; Li, H. Improved Electrochemical Performances of High Voltage $LiCoO_2$ with Tungsten Doping. *Chin. Phys. B* **2018**, *27*, 088202, doi:10.1088/1674-1056/27/8/088202.
